# Interplay Between Macrophages and Angiogenesis: A Double-Edged Sword in Liver Disease

**DOI:** 10.3389/fimmu.2019.02882

**Published:** 2019-12-12

**Authors:** Marta Ramirez-Pedraza, Mercedes Fernández

**Affiliations:** ^1^Angiogenesis in Liver Disease Research Group, IDIBAPS Biomedical Research Institute, Hospital Clinic, University of Barcelona, Barcelona, Spain; ^2^Biomedical Research Networking Center on Hepatic and Digestive Disease (CIBEREHD), Institute of Health Carlos III, Madrid, Spain

**Keywords:** angiogenesis, macrophages, Kupffer cells, hepatic stellate cells, vascular endothelial growth factor

## Abstract

During chronic liver disease, macrophages support angiogenesis, not only by secreting proangiogenic growth factors and matrix-remodeling proteases, but also by physically interacting with the sprouting vasculature to assist the formation of complex vascular networks. In the liver, macrophages acquire specific characteristics becoming Kupffer cells and working to ensure protection and immunotolerance. Angiogenesis is another double-edged sword in health and disease and it is the biggest ally of macrophages allowing its dissemination. Angiogenesis and fibrosis may occur in parallel in several tissues as macrophages co-localize with newly formed vessels and secrete cytokines, interleukins, and growth factors that will activate other cell types in the liver such as hepatic stellate cells and liver sinusoidal endothelial cells, promoting extracellular matrix accumulation and fibrogenesis. Vascular endothelial growth factor, placental growth factor, and platelet-derived growth factor are the leading secreted factors driving pathological angiogenesis and consequently increasing macrophage infiltration. Tumor development in the liver has been widely linked to macrophage-mediated chronic inflammation in which epidermal growth factors, STAT3 and NF-kβ are some of the most relevant signaling molecules involved. In this article, we review the link between macrophages and angiogenesis at molecular and cellular levels in chronic liver disease.

## Introduction

The liver is both the largest organ of the body and the largest gland, weighing about 1.5 kg. It is situated in the abdominal cavity beneath the diaphragm. It carries out more than 500 essential roles having an impact in both physiology and disease (1). The major functions of the liver may be summarized as follows: Detoxification of metabolic waste products; destruction of spent red cells; and reclamation of their constituents (in conjunction with the spleen); synthesis and secretion of bile into the duodenum via the biliary system; synthesis of the plasma proteins including the clotting factors but excluding the immunoglobulins; synthesis of plasma lipoproteins; and metabolic functions, e.g., glycogen synthesis and gluconeogenesis. Many of these biosynthetic functions directly utilize the products of digestion. With the exception of most lipids (which are transported mainly by lymph vessels), absorbed food products pass directly in the venous blood from the small intestine to the liver via the portal vein before entering the general circulation. Thus, the vascular bed of the liver is perfused by blood rich in amino acids, simple sugars, and other products of digestion but relatively poor in oxygen. Oxygen required to support the intense metabolic activity of the liver is supplied in the arterial blood via the hepatic artery. The liver, therefore, is unusual in that it has a dual blood supply that is both arterial (20%) and venous (80%). Venous drainage of the liver occurs *via* the hepatic vein and lymph from the liver is drained directly into the thoracic duct. The position of the liver in the circulatory system is therefore optimal for gathering, transforming, and accumulating metabolites and for neutralizing and eliminating toxic substances. This elimination occurs in the bile, an exocrine secretion of the liver that is important in lipid digestion.

The microanatomy of the liver is key for the achievement of the multifaceted hepatic abilities and homeostasis maintenance. The principal and most abundant cells of the liver, the hepatocytes, are arranged into polygonal lobules, the structure of which maximizes contact of hepatocytes with blood flowing through the liver. At the corners of the lobules, there are portal triads, each with a venule (a branch of the portal vein), an arteriole (a branch of the hepatic artery), and a duct (part of the bile duct system). The hepatocytes are radially disposed in the liver lobule. They form a layer of one or two cells thick, arranged like the bricks of a wall. The space between these cellular plates contains the liver sinusoids, composed solely of a discontinuous layer of fenestrated liver sinusoidal endothelial cells (LSECs) (2, 3). The sinusoids arise in the periphery of the lobule, fed by the terminal branches of portal veins and hepatic arterioles at the portal triads, and run in the direction of the hepatic central vein. The endothelial cells are separated from the underlying hepatocytes by a subendothelial space known as space of Disse, which contains microvilli of the hepatocytes. Blood fluids readily percolate through the endothelial wall and make intimate contact with the hepatocyte surface, permitting an easy exchange of macromolecules from the sinusoidal lumen to the liver cell and vice versa. This is physiologically important not only because of the large number of macromolecules (e.g., lipoproteins, albumin, fibrinogen) secreted into the blood by hepatocytes but also because the liver takes up and catabolizes many of these large molecules. In addition to the LSECs, the sinusoids contain phagocytic cells known as Kupffer cells (KCs) (3). The main functions of these hepatic macrophages are to metabolize aged erythrocytes and other particulate debris from the circulation, digest hemoglobin, and secrete proteins related to immunologic processes. The hepatic stellate cells (HSCs), located in the space of Disse, have the capacity to accumulate exogenously administered vitamin A as retinyl esters in lipid droplets (4, 5).

**Figure 1:**
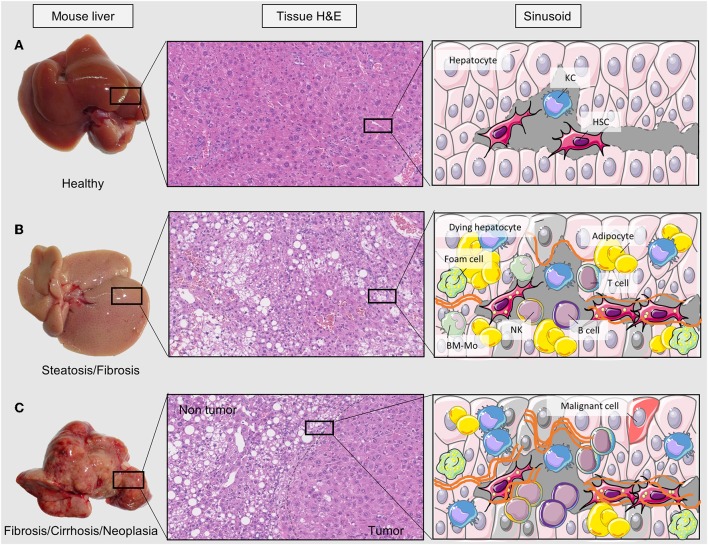
Liver disease stages and tissue alterations. Changes in liver tissue can be detected macroscopically and microscopically in this figure. There are three sets of pictures; **(A)** healthy tissue, **(B)** fibrotic liver, and **(C)** tumorigenic. At the left side of the three sets there are pictures of livers extracted from animal models in our research group. In the next column there are hematoxylin and eosin staining pictures of those tissues where hepatocytes (pink) and adipocytes (round and white) can be seen. At the sinusoid column there is a scheme of tissue cell infiltration and hepatocyte transformation into dying hepatocytes or tumor cells. H&E, hematoxylin eosin staining; KC, Kupffer cell; HSC, hepatic stellate cell; T cell, lymphocyte T; NK, natural killer cell; B cell, lymphocyte B; BM-Mo, bone marrow derived macrophage.

Liver disease comprises different disease stages and is mainly caused by obesity, alcohol consumption, diabetes, or viral infections (6). Non-alcoholic fatty liver disease (NAFLD) and alcoholic fatty liver disease (AFLD) only differ on the etiology; they are the first stages of disease and consist on the accumulation of triglycerides within hepatocytes. This excessive accumulation impairs hepatocyte functionality and promotes tissue inflammation driving toward non-alcoholic steatohepatitis (NASH) development (7). Activation of the immune component and other cellular types such as HSCs and LSECs promotes extracellular fiber deposition (collagen and other matrix constituents) and thus liver fibrosis that will progress toward the next stage of liver disease—cirrhosis—if inflammatory signals remain overexpressed. Hepatocellular carcinoma (HCC) can grow in livers affected by all the etiologies, but it is usually the last stage of disease after cirrhosis (8) (Figure 1).

## Macrophages in the Liver

Macrophages are myeloid immune cells with the ability to phagocyte pathogens, dead cells, cellular debris, and various components of the extracellular matrix. Ilya Ilyich Mechnikov was the first to describe the process of phagocytosis in 1882, and macrophages were named after this feature as “big eaters” (from ancient Greek, makros “large” + phagein “eat”). However, it is now clear that macrophages are not only big eaters of pathogens and dead cells, but also important components of the stromal architecture of several tissues and organs, where they regulate organ homeostasis and remodeling. For instance, KCs are specialized macrophages that line hepatic sinusoids in the liver, where they scavenge senescent erythrocytes, a process referred to as hemocatheresis (9). During development and tissue healing or regeneration, macrophages stimulate angiogenesis, and facilitate tissue remodeling by secreting a number of proteases and growth factors (9).

**Figure 2:**
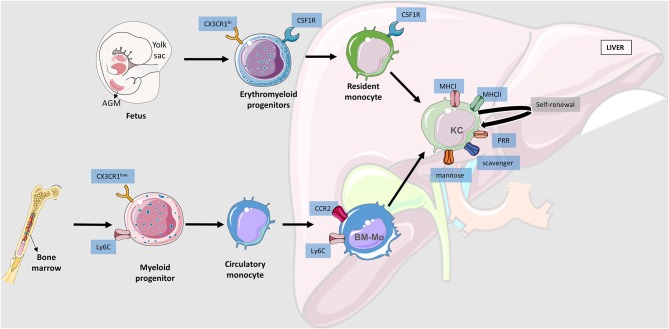
Liver macrophages origin. Schematic representation of macrophages' movement toward the liver. There are three main niches where hematopoietic stem cells evolve and become myeloid progenitors: aorta–gonad–mesonephros (AGM), yolk sac (YS), and bone marrow. YS and AGM derived progenitors are generally considered the same due to their similarities. Erythromyeloid progenitors will go to the fetal liver and become resident monocytes that will evolve into Kupffer Cells (KC) establishing the liver resident macrophage population with self-renewal ability. Bone marrow produced myeloid progenitors that go to the systemic circulatory system and remain there until injury signals activate the cascades for tissue infiltration. Bone marrow derived macrophages (BM-Mo) require CCR2 (C-C chemokine receptor type 2) to be able to infiltrate into the liver and may undergo a conversion into a KC if necessary.

The liver bears the biggest proportion of macrophages among all solid organs in the body. The full spectrum of immune cells in the liver is not yet totally clear, but populations of liver-resident macrophages such as KCs have been well-characterized. They present pattern recognition receptors (PRR) for the detection and degradation of microbial-associated molecular patterns (MAMP) and damage-associated molecular patterns (DAMP) (10). KCs in the liver may have different origins: yolk sac, bone marrow or hematopoietic stem cells derived from the ventral wall of the aorta in the aorta–gonad–mesonephros (AGM) region (11) (Figure 2). Erythromyeloid progenitors from the yolk sac express macrophage colony-stimulating factor 1 receptor (CSF1R) and allow differentiation into KC in the fetus during development (12). Bone marrow derived macrophages CCR2^+^ (C-C chemokine receptor type 2) LY6C^+^ (lymphocyte antigen 6 complex) can be recruited into the liver and achieve KC-like phenotype. Monocyte recruitment happens mostly after liver injury and under inflammatory conditions as a response to reestablish tissue homeostasis, and when they are excessive they undergo apoptosis (13). Close to week 5 of fetus gestation, hematopoietic stem cells derived from the AGM colonize the liver and give rise to mature erythroid, lymphoid and myeloid cells. Due to the similarities between yolk sac and AGM derived macrophages present in the liver, most studies consider them as the same (13).

Depending on the cell origin and the differentiation process, macrophages acquire different phenotypic (surface marker profile and gene expression) and functional features, which are variable between mice and humans. For instance, CD14^++^CD16^−^ classical human monocytes or intermediate CD14^++^CD16C^+^ monocytes correspond to GR1^+^/Ly6C^high^ inflammatory monocytes in the mouse and are CCR2^+^Cx3CR1^low^ (14). Cx3CR1, also called fractalkine receptor, is considered a key regulator of macrophage activity (15).

In addition to KCs, other subsets such as monocyte-derived macrophages, myeloid dendritic cells, scar-associated macrophages, inflammatory macrophages, restorative macrophages, tumor-associated macrophages, and monocytic myeloid-derived suppressor cells can be found in the liver. The acquisition of a pro-inflammatory or anti-inflammatory phenotype depends on the local tissue microenvironment (9, 10, 16). Knowing the full spectrum of macrophage activation, the underlying molecular mechanisms, and their implication in either promoting liver disease progression or repairing injured liver tissue is highly relevant from a therapeutic point of view. For instance, scar-associated macrophages positively contribute to fibrosis resolution by producing chemokine (C-X-C motif) ligand 9 and matrix metalloproteinase 13 (17). Other studies have shown the importance of specific receptors involved in macrophage activation such as integrin alphavbeta 3. This is a receptor for vitronectin, and its inhibition *in vivo* decreases angiogenesis and worsens liver fibrosis outstanding the complexity of therapeutic strategies required for patients with liver disease (18). It should also be taken into consideration that animal research, particularly that relating to phagocyte and immune networks, may be poor predictors of human pathophysiology (19).

## Pathophysiology of Liver Angiogenesis

Angiogenesis is the process of new vasculature generation from pre-existing blood vessels and is present in health and disease. It is a tightly regulated process as excessive angiogenesis may prelude the establishment of abnormal vasculature and thus disease promotion (20, 21). Little is known about the mechanisms by which the endothelial cells present at the leading edge of vascular sprouts (named endothelial “tip” cells) integrate directional cues from the environment and fuse to form new functional blood vessels. In the pathological setting of chronic liver disease, angiogenesis has been related to progressive liver inflammation, fibrogenesis, and tumorigenesis (1, 22, 23). Pathological angiogenesis in liver disease also occurs extrahepatically, playing a major role in the formation of porto-systemic collateral vessels and the development and aggravation of splanchnic hemodynamic disturbances and portal hypertension (1, 23). Angiogenesis is also an essential hallmark in liver cancer, allowing not only tumor nourishment and thus growth but also its dissemination toward other organs (23).

Vascular endothelial growth factor (VEGF), placental growth factor (PlGF), and platelet derived growth factor (PDGF) are the leading secreted factors driving pathological angiogenesis in liver disease (1, 23). Increasing the supplying blood vessels of the liver can, in turn, further augment the recruitment of inflammatory cells, which will stimulate inflammation and activate profibrogenic myofibroblasts thereby resulting in fibrogenesis. In addition, the abnormally formed new vessels are very different from the highly specialized intrahepatic sinusoidal vessels. Thus, they are disorganized, chaotic, leaky vessels, which, instead of improving the effective perfusion of hepatocytes, they further compromise the oxygen and nutrient delivery to the liver parenchyma, resulting in further hepatocyte damage and hypoxia. This will exacerbate myofibroblast activation and fibrogenesis and will impair the effectiveness of inflammatory response. Moreover, proangiogenic factors can activate myofibroblasts not only through angiogenesis, but also by a direct activation of these cells, which express receptors for proangiogenic factors. Activated myofibroblasts are also able to produce proangiogenic factors, which further facilitate their own transdifferentiation and also mediate specialized cellular functions such as proliferation, chemotaxis, and production of extracellular matrix. Accordingly, inhibition of angiogenesis, for example, using the multikinase inhibitor sorafenib, causes a marked decrease in the intrahepatic neovascularization, fibrosis, and inflammation observed in animal models of cirrhosis (24).

## Mechanisms Linking Macrophages and Angiogenesis

### Cellular

Macrophages likely represent the preeminent cells in the body endowed with the ability to migrate within tissues, even in hypoxic conditions, and with the capacity to modify the extracellular matrix and amplify paracrine signals. Macrophages may thus provide temporary scaffolds or paracrine support for the expansion and maturation of vascular networks, both in development and in pathophysiological conditions.

In liver disease, the main producers of pro-angiogenic factors are HSCs, portal fibroblasts, and myofibroblasts (25, 26). Activation of these cells toward angiogenesis promoters is usually stimulated by hypoxia inducible factor 1 (HIF-1α), but the presence of both infiltrated and resident macrophages also contributes to generate the required vascular growth factor signals. Moreover, endothelial cells also participate in the establishment of a pro-angiogenic microenvironment by leptin secretion easing the stabilization of newly formed vessels in a context of advanced fibrosis where fibrotic septums are more mature (25).

Activated HSCs act, through the expression of VEGF receptor and VEGF family factors, in an autocrine and paracrine manner thus having an impact on LSEC and aggravating fibrosis. VEGFR2 (vascular endothelial growth factor receptor) modulates the pro-fibrogenic activity of HSC and LSECs and, subsequently, angiopoietin-1 activates Tie-2 tyrosine-protein kinase receptors (epidermal growth factor homology-2) on LSEC for vessel stability. The role of LSECs in the development of liver disease is tightly associated with VEGF secreted by hepatocytes and HSC as it determines their fenestrated phenotype that will also have an impact on HSC activation when it becomes abnormal (26).

Differently polarized macrophages have been characterized and associated with several processes involved in liver disease. Macrophages derived from bone marrow are CCR2^+^ and Ly-6C^+^ and require CCL2 to infiltrate into the liver (27). They play an essential role in angiogenesis regulation as ablation of CCL2 prevented angiogenesis associated with fibrosis, although it does not affect fibrosis development. Myofibroblasts are rather like macrophages as they also present heterogeneous populations in the liver which include HSC, derived cells like periportal fibroblasts, and epithelial-to-mesenchymal transition cells, all of them with different angiogenic capabilities (28).

Kupffer cells are constantly surveilling the liver, being responsible for the removal of damaged red cells from the blood circulation. This process is mediated by polyinosinic acid- and phosphatidylserine-sensitive scavenger receptors, different from scavenger receptor class A type I and II (29, 30). Kupffer cells require the recruitment of additional immune cells to carry out microbial and antigen total clearance. These other immune components are lymphocytes B (B cell) and T (T cell) as well as natural killers (NK) and other types of lymphocytes that are distributed all over the liver parenchyma (31).

Interestingly, the relationship between macrophages and vessels can be carried back to the hematopoietic processes taking place during fetal development and vascular patterning. Liver is one of the most important hematopoietic organs, and this fact becomes of maximum relevance in fetal liver when it becomes a niche for hematopoietic stem cells. Although the signaling pathways switching on maturation or migration programs are yet to be established, an intimate proximity has been set between those hematopoietic stem cells that will give rise to macrophages and pericytes within portal vessels (32).

### Molecular

**Figure 3:**
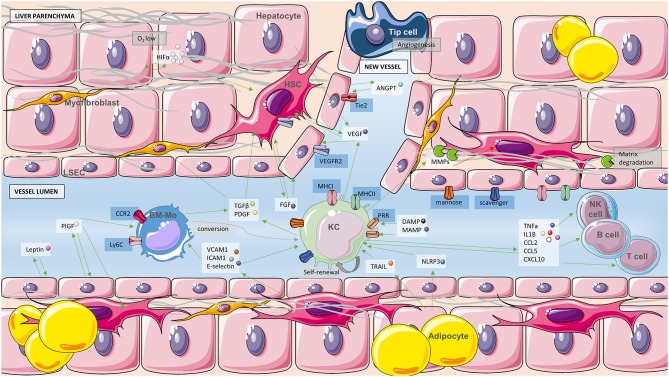
Angiogenesis and macrophages. Schematic representation of the interplay between immune cells and the process of angiogenesis. Green arrows indicate activation of cells or secretion of soluble molecules and red arrows inhibition or inactivation. Soluble molecules are in white boxes, blue boxes refer to receptors and gray boxes to processes. Cell types present in the diagram consist of hepatocytes, myofibroblasts, hepatic stellate cells (HSC), liver sinusoidal cells (LSEC), adipocytes, Kupffer cells (KC), bone marrow-derived macrophages (BM-Mo), lymphocytes NK, T, and B as well as one tip cell leading to angiogenesis. Long gray structures are fibers of collagen. MMPs, metalloproteinases; ANGPT, angiopoietin; CCL2, C-C motif chemokine ligand 2; CCL5, C-C motif chemokine ligand 5; CXCL10, C-X-C motif chemokine ligand 10.

Interestingly, most of the molecules involved in angiogenesis are also involved in inflammation and the other way around. In fact, macrophages derived from the bone marrow are internalized into the liver parenchyma from the vasculature and mature, losing LY6C surface marker. Once there, they achieve the ability to degrade extracellular matrix secreting metalloproteinases (MMP) and become antifibrotic macrophages decreasing HSC activity (33). They can also produce VEGF-A and induce vessel restoration and phagocytosis of dead cells as well as extracellular matrix restoration after acute liver injury (34). Both resident and infiltrating macrophages possess profibrogenic abilities secreting transforming growth factor beta (TGF-β) and PDGF and activating HSCs and myofibroblasts (35). After injury, other cytokines such as tumor necrosis factor alpha (TNF-α) and interleukin 1 beta (IL-1β) and chemokines (CCL-2, CCL5 and CXCL10) are usually secreted by macrophages exacerbating inflammation and boosting angiogenesis (36). Furthermore, KC and bone marrow-derived macrophages can be recruited by HSC secretion of adhesion molecules such as vascular cell adhesion molecular-1 (VCAM-1), intracellular adhesion molecule 1 (ICAM-1), and E-selectin (37) (Figure 3). In addition to expressing classic proangiogenic and tissue-remodeling factors, which may initiate angiogenesis, macrophages appear to support the formation of a functional vascular system by (i) assisting directional vessel growth *via* cell-to-cell contacts and/or their production of guidance factors that act iuxtacrinally on vascular sprouts after the induction of endothelial cell proliferation and angiogenesis; (ii) pruning primitive blood vessels (via secretion of proapoptotic factors) to remodel the vascular network.

The difficulties in targeting angiogenesis reside in the ability of cells to activate compensatory pathways or even the physiological presence of redundant pathways. In this respect, several research groups have shown the relevance of PlGF, a member of the VEGF family involved in endothelial cell and bone marrow-derived cell activation, pathologic angiogenesis, and inflammation (38). PlGF is expressed by macrophages, ECs, and HSCs and specifically binds to VEGFR1. It is upregulated in fibrosis, cirrhosis, and hepatocellular carcinoma (39). Silencing PlGF in mice reduces tumor associated macrophage (TAM)-related chemokines and receptors (CXCL10, ICAM-1, VCAM, and CCR2), pro-inflammatory molecules (TNF-α, IL-1β, CCL2), and even anti-microbial receptors (TLR4 and TLR9), emphasizing the crucial role of this molecule and thus its potential as a therapeutic target (40). Another family of growth factors that may be involved in therapeutic failure is fibroblast growth factors (FGF) which are involved in angiogenesis and inflammation and essential for resolving liver regeneration (41).

## Impact of Macrophages and Angiogenesis on Disease Progression

Interestingly, liver macrophages derived from circulating monocytes, which invade the tissue after injury, colocalize with newly formed vessels. They present pro-angiogenic genetic profiles with overexpression of VEGF and MMP9 and are mostly found in portal tracts (27). These macrophages require CCL2, linking angiogenesis with inflammation. Indeed, inhibition of CCL2 reduced angiogenesis and monocyte infiltration at the beginning of fiber accumulation in the liver, indicating that there is a tight correlation between the extent of fibrosis and the recruited inflammatory components. These results indicate that even though angiogenesis is dependent on macrophage infiltration at the first disease stages, this does not attenuate fibrosis progression. However, when disease progresses toward chronic and even neoplasia development, angiogenesis becomes increasingly a cause for disease progression rather than a consequence (27, 42). The role of macrophages in disease development has been proven to be essential as its depletion causes disease development attenuation in both NAFLD and ALD in animal models (43).

### Impact on Steatosis and Fibrosis

Chronic liver disease is characterized by inflammatory and fibrogenic processes that involve pathological angiogenesis, and depending on the origin and etiology of the fibrogenic process development, angiogenesis will have a variable impact on disease progression and reversibility (44). Angiogenesis usually occurs in parallel to fibrosis, and microvessel density has been correlated with the degree of accumulated fiber and vice versa. Even more, liver angiogenesis is different from the same process taking place in other organs or tissues and has intrinsic angiogenic factors such as ANGPTL3 (angiopoietin-like 3) (45). The convergence of increased tissue hypoxia due to fiber deposition and anatomical rearrangement of liver tissue together with wound healing processes that try to reestablish tissue homeostasis generates a characteristic environment full of metalloproteinase, growth factors (PDGF, TGF-1β, FGF, VEGF), cytokines, and adhesion molecules that will promote angiogenesis and fibrosis and settle the perfect conditions for disease chronicity (6).

In the presence of lipid accumulation in the liver and thus in the context of NAFLD, hepatocytes secrete vesicles containing TNF-related apoptosis inducing ligand (TRAIL) among other molecules, and they stimulate macrophages polarizing KC toward inflammation promoters. Conversely, they can switch and trigger apoptosis and autophagy of inflammatory cells ameliorating inflammation and hence fibrosis development (46). Experimental mice models suggest that during non-alcoholic steatohepatitis (NASH) the need for rapid lipid drop through metabolism and translocation gives rise to infiltrated macrophages in the first place instead of KC. Apparently, when injury stimulation remains constant and disease progresses toward chronic liver disease, then resident macrophages play the main role.

In this perspective a tight relationship has been established between macrophages, lipid metabolism, and hepatic steatosis progression. The link has been made through NOD-, LRR-, and pyrin domain-containing 3 (NLRP3) inflammasome (47) which activates pathways that allow an alternative polarization in macrophages. This specific polarization is related to the activation of steatogenic signaling in hepatocytes and altogether assemble intricate and redundant pathways in which metabolism regulates macrophages and macrophages regulate metabolism (48).

In fibrosis, macrophages play a dual role as their presence increases scarring but at the same time is required for proper tissue repair. Infiltrating macrophages accumulate in the liver and, together with KC, secrete factors, such as TGFβ and PDGF that promote survival and activation of HSC thus acting as profibrogenic cells. This capability is achieved by the influence of NKT cells and other immune components together with factors present in the microenvironment (49).

### Impact on Cirrhosis

When liver fibrosis remains constant and other conditions such as obesity, diabetes, malnutrition, and alcoholism contribute to fiber accumulation and chronic inflammation, then liver fibrosis becomes liver cirrhosis which is the next and more severe stage in terms of loss of liver functionality and usually preludes development of hepatocellular carcinoma (50). The presence of macrophages is increased in cirrhosis, similarly observed in liver fibrosis. However, in this advanced disease stage, the need for toxin clearance is dangerously elevated and the state of immunocompetency can move toward an immunodeficiency when cirrhosis is decompensated and gut permeability increases together with PAMP exposure, increasing the risk of mortality (51). The immune system is extremely activated and cytokines are overexpressed in cirrhosis. Distribution and functionality of monocytes are altered; they are mostly pro-inflammatory expressing CD14^+^CD16^+^, and their phagocytic activity is limited (52).

In cirrhosis, the liver becomes highly hypoxic and angiogenesis is stimulated to compensate the lack of oxygen and nutrients (53). Signals triggering the angiogenesis cascade include HIF-1α, which upregulates the expression of the angiogenic growth factor VEGF. Interestingly, HSCs acquire an angiogenic phenotype stimulated by platelet-derived growth factor (PDGF). This angiogenic phenotype is characterized by enhanced HSC-driven vascular tube formation *in vitro* and enhanced HSC coverage of sinusoids *in vivo* (54). HSC activation becomes excessive because of NO deficiency in cirrhotic livers and consequently, liver perfusion is compromised (55). Increased angiogenesis in portal areas, associated with enhanced inflammatory microenvironment, has been observed in patients with primary biliary cirrhosis (56).

### Impact on Tumorigenesis

**Figure 4:**
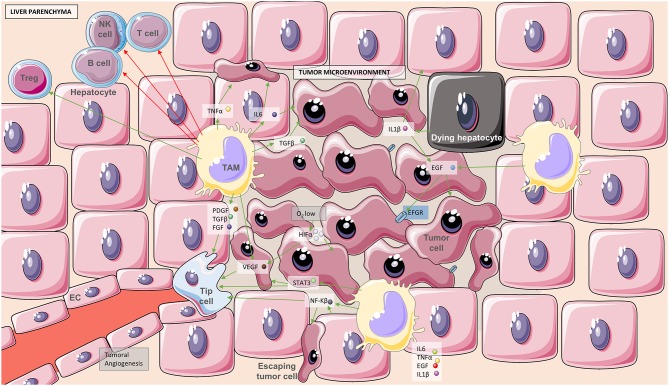
Tumor microenvironment boosts angiogenesis and inflammation. Schematic representation showing the interplay between tumor cells and the processes of immune cell infiltration and angiogenesis. Hypoxic regions in the tumor microenvironment generate the adequate signaling cascades to activate angiogenesis and thus be able to supply tumor cells with the required nutrients. Green arrows indicate activation of cells or secretion of soluble molecules and red arrows inhibition or inactivation. Soluble molecules are in white boxes, blue boxes refer to receptors, and gray boxes to processes. Cellular components consist of hepatocytes, dying hepatocytes, tumor cells, escaping tumor cells, tumor associated macrophages (TAM), endothelial cells (EC), lymphocytes NK, T, and B as well as one tip cell leading to angiogenesis. IL, interleukin.

Hepatocellular carcinoma is the most common type of primary liver cancer and the third leading cause of death related to cancer in the world. Macrophages contribute to growth, angiogenesis, and metastasis in hepatocellular carcinoma (57, 58). Although macrophages resolutely contribute to tumor surveillance they generally become tumor associated macrophages (TAM), which are highly pro-inflammatory, and provide the necessary signals to create a pro-tumorigenic environment and to inhibit immune responses against it (59) (Figure 4). This malignant conversion occurs in both KC and infiltrated macrophages. In this context, not only pro-tumorigenic signals are favored by macrophages, but also pro-angiogenic factors such as VEGF, PDGF, TGFβ, and FGF, which allow tumor growth establishment and expansion. TAMs have the ability to be polarized toward pro-inflammatory or anti-inflammatory phenotypes. Essentially, interleukin 6 (IL6) and TGFβ promote tumor growth, IL6 together with TNFα facilitates invasion and metastasis, and TGFβ with IL10 suppresses the immune response against the tumor. But not only this, they even have the ability to activate T helpers type 2 (Th2) and thus recruitment and activation of regulatory T cells (Tregs) which are often involved in self-tolerance (58, 60).

Macrophages associated with hepatocellular carcinoma have a high expression of epidermal growth factor receptor (EGFR), which induces IL6 signaling pathway, and hepatocyte proliferation being thus considered a tumor-promoting factor. IL6 is usually expressed after injury as a response to IL1β derived from dying hepatocytes. However, EGFR-expressing macrophages have also been found in tissues surrounding tumors, indicating a higher level of cirrhosis and poor prognosis (61, 62). The NF-κB signaling pathway plays an important role in linking liver inflammation with cancer (63, 64). In response to pro-inflammatory signals such as TNF or IL1β, IκB kinases (IKK) that have NF-κB kidnapped in the cytoplasm are degraded leaving NF-κB (nuclear factor kappa-light-chain-enhancer of activated B cells) free (64). It is mainly produced by hepatocytes but Kupffer cells can also induce its activation and it has been related to tumor progression (65). Another transcription factor involved in HCC development and bad prognosis is STAT3 (signal transducer and activator of transcription 3) (63). It is activated by inflammatory signals such as IL6 and epidermal growth factor (EGF) or reactive oxygen species (ROS), and it is expressed by macrophages to prevent chronic inflammation (63). However, once the disease is established, STAT-3 promotes oncogenesis through the Src oncogene (66). Both NF-κB and STAT3 interact to promote tumor growth by inducing activation of pathways related to angiogenesis, hypoxia, chemokines, and immunosuppression.

Angiogenesis is a process whereby new vessels sprout and branch from preexisting blood vessels. Mechanisms for physiological and tumor related angiogenesis are similar but the consequences are far from alike. While physiological angiogenesis allows a homeostatic balance, tumor derived angiogenesis offers tumor cells the ability to survive, propagate, and invade other tissues. In cancer, the new vasculature is structurally and functionally abnormal; blood vessels are immature and leaky (67, 68). Tumor angiogenesis is basically a four-step process: First, the basement membrane in tissues is injured; second, endothelial cells, activated by angiogenic factors, migrate; third, endothelial cells proliferate and stabilize; and four, angiogenic factors continue to influence the angiogenic process. Several studies indicate that the levels of angiogenic factors, mainly VEGF, reflect the aggressiveness of tumor cells and thus have a predictive value in the identification of the high-risk patients with poor prognosis. Angiogenic factors are also attractive therapeutic targets for hepatocellular carcinoma (69, 70). Hypoxia and nutrient deprivation trigger the process of neovessel formation by inducing tumor cells to release soluble pro-angiogenic growth factors, chemokines, and cytokines (67, 68). In addition, the tumor microenvironment, including tumor-induced inflammatory responses, recruits multiple cell types and releases the stimulus required to support angiogenesis and allow tumor progression. These immune cells are an important source of matrix metalloproteinase to degrade ECM and promote cell invasion.

Tumor evasion against anti-angiogenic therapy might be propitiated by five mechanisms (71). First, tumor heterogeneity might lead to the co-existence of diverse angiogenic growth factors that can be positively selected in case of a treatment with a single pathway inhibitor. Second, due to therapy, a genetic switch and overexpression of other pro-angiogenic factors can occur. Even more, hypoxic regions can increase and thus upregulate angiogenic growth factors. Another factor to be considered is compensatory programs, which are very coordinated in response to homeostasis perturbation. An example of this is the upregulation of VEGF receptor-2 signaling with the silencing or lack of expression of β3 or β5 integrins; if integrins and VEGF promote maximal signal transduction downstream of the angiogenic pathways, disabling these interactions by impairing both components might therefore be subjected to less compensation or resistance (71).

### Impact on Extrahepatic Complications Related to Liver Disease

The crosstalk between angiogenesis and macrophages also plays a role in liver disease-related complications taking place outside the liver such as in splanchnic organs, contributing to progression and aggravation of the pathology. Macrophages and angiogenesis contribute to fibrosis development *via* gut-liver axis activation as both Kupffer cells and HSCs become active through TLR receptors which are dependent on the presence of endotoxins derived from the gut (72). Metabolic disorders contribute to intestinal dysbiosis that leads to gut-vascular barrier leakage and foreign bodies entering the blood stream toward the liver. This process is called bacterial translocation and causes systemic inflammation, macrophages being essential players in both cause and resolution (73). In many cases, liver disease derives from obesity or other metabolic alterations. Fat accumulation in visceral fat depots, including the mesentery, induces a state of chronic inflammation that may reach the liver through the portal venous system, contributing to activate HSCs and increase fibrogenesis (74). Pathological angiogenesis during chronic liver disease also takes place extrahepatically, playing a major role in the formation of portosystemic collaterals (1, 75–80) and the development and maintenance of splanchnic hyperdynamic circulation and portal hypertension (81–85).

## Targeting Angiogenesis-Inflammation Crosstalk in Liver Disease

As mentioned above, inhibition of angiogenesis is at the same time beneficial and damaging, and therapies should ideally affect only pathological angiogenesis leaving the basal level required for wound healing, tissue repair, and other physiological functions of angiogenesis. Due to the relevance of VEGF as a key angiogenic regulator, it is one of the main targets. Approaches used to inhibit angiogenesis in chronic liver disease and portal hypertension include neutralizing monoclonal antibodies (78), tyrosin kinase inhibitors (24, 83–85), or therapeutic small interference RNAs targeting VEGF receptor-2 (76). Attenuation of oxidative stress and inflammation has also been shown to reduce pathological angiogenesis in liver disease and portal hypertension (86–88). Promising results with anti-VEGF therapy have also been demonstrated in hepatocellular carcinoma (69, 89). Another interesting strategy to inhibit pathological angiogenesis is by therapeutically increasing the expression of angioinhibitors that are endogenously present in the body, such as pigment epithelium derived factor (PEDF) or vasohibin (90, 91). A prominent advantage of using these natural inhibitors is that they would not be expected to activate drug resistant genes and thus may offer a promising breakthrough for effective antiangiogenesis therapy. Recent studies also highlight the functional significance of pathologic neovascularization derived from vascular stem/progenitor cells as an important mechanism of formation of new blood vessels in adults, in the setting of chronic liver disease, and identify these stem cells as potential new therapeutic targets (92). In a search for ways to inhibit pathologic production or activities of VEGF without affecting its normal production or functions, our research group has investigated the post-transcriptional regulation of VEGF by the cytoplasmic polyadenylation element-binding proteins CPEB1 and CPEB4 during development of liver disease (93, 94). We have identified a mechanism of VEGF overexpression in the liver and mesentery that promotes pathologic, but not physiologic, angiogenesis, via sequential and non-redundant functions of CPEB1 and CPEB4. Activation of CPEB1 promotes alternative nuclear processing within non-coding 3′-untranslated regions of VEGF and CPEB4 mRNAs, resulting in deletion of translation repressor elements. The subsequent overexpression of CPEB4 promotes cytoplasmic polyadenylation of VEGF mRNA, increasing its translation and generating high levels of VEGF protein, which induces pathologic angiogenesis in chronic liver disease. From a translational point of view, our studies highlight that CPEBs could be promising angiogenesis-disrupting targets in disease. Thus, targeting CPEBs could lead to safer treatment outcomes by specifically reducing excessive pathological VEGF production instead of indiscriminately perturbing both pathological and physiological VEGF synthesis, minimizing potential adverse side-effects. Reduction of pathological angiogenesis in early disease stages could also prevent further disease progression and reduce the risk for developing overt liver cirrhosis. Accordingly, development and evaluation of CPEB inhibitors are currently underway. As better and more specific inhibitors of pathologic angiogenesis are developed, combination strategies continue to evolve, and increased understanding of the complex biology of angiogenesis takes place, antiangiogenic therapy will certainly be evaluated in future clinical trials.

## Conclusion

The interplay between macrophages and angiogenesis determines the progression of a big number of diseases but, in the liver, this is especially important due to the particularities of the processes of vasculogenesis and inflammation that are taking place. Liver vasculature and microvasculature are essential not only for tissue reoxygenation but also for it to work as the main filter between toxins and the rest of the body, and to accomplish that function the immune system has to maintain its ability to switch from immuno-tolerant to responsive constantly. Essentially, both processes require the presence of the other and when cellular stress remains for a period of time and homeostasis is lost, they boost each other through the detection and secretion of common signaling molecules. For that, it is mandatory to go further in research to decipher more mechanisms that would allow to therapeutically target pathologic levels of both processes without inhibiting immune surveillance and tissue regeneration capabilities. Currently, although there are some drugs approved for all stages of liver disease, there is always the risk of treatment failure due to redundant mechanisms, which claims the need for the detection of specific targets or pathways to avoid the “double-sword” effect.

## Author Contributions

MR-P: drafting of the manuscript and preparation of figures. MF: review concept, design, and supervision, drafting of the manuscript, and funding.

### Conflict of Interest

The authors declare that the research was conducted in the absence of any commercial or financial relationships that could be construed as a potential conflict of interest.
